# Growth and structure analysis of tungsten oxide nanorods using environmental TEM

**DOI:** 10.1186/1556-276X-7-85

**Published:** 2012-01-25

**Authors:** Tomoharu Tokunaga, Tadashi Kawamoto, Kenta Tanaka, Naohiro Nakamura, Yasuhiko Hayashi, Katsuhiro Sasaki, Kotaro Kuroda, Takahisa Yamamoto

**Affiliations:** 1 Department of Quantum Engineering, Nagoya University, Furo-cho, Chikusa-ku, Nagoya, Aichi, 464-8603, Japan; 2 Department of Frontier Materials, Nagoya Institute of Technology, Gokiso-cho, Showa-ku, Nagoya, Aichi, 466-8555, Japan

**Keywords:** tungsten, oxide, nanorod, environmental TEM

## Abstract

WO_3 _nanorods targeted for applications in electric devices were grown from a tungsten wire heated in an oxygen atmosphere inside an environmental transmission electron microscope, which allowed the growth process to be observed to reveal the growth mechanism of the WO_3 _nanorods. The initial growth of the nanorods did not consist of tungsten oxide but rather crystal tungsten. The formed crystal tungsten nanorods were then oxidized, resulting in the formation of the tungsten oxide nanorods. Furthermore, it is expected that the nanorods grew through cracks in the natural surface oxide layer on the tungsten wire.

## Background

Metal oxides such as ZnO, In_2_O_3_, and WO_3 _are well known as bandgap semiconductors, which led to the development of many growth methods. During the studies into these growth methods, nanoscale metal oxides were discovered. These nanoscale materials have been widely studied since the electronic characteristics of nanoscale materials are different from those of bulk-scale materials [1-5]. In particular, metal oxides with nanorod structures were studied because they have a one-dimensional structure and are thus able to be applied for electrical components such as nanoscale wires. Tungsten oxide nanorods are one of the metal oxide semiconductors that can be easily made [6-8]. Therefore, due to its semiconducting properties, it is applied in electrical devices. However, the growth mechanism of tungsten oxide nanorods has not yet been clarified, and the growth of tungsten oxide nanorods has not been successfully controlled. In this study, the tungsten oxide nanorod growth process was observed using an environmental transmission electron microscope [TEM], and the growth mechanism was examined.

## Methods

The growth of tungsten oxide nanorods was conducted by heating a tungsten wire in an oxygen atmosphere inside an environmental TEM. The commercially obtained pure tungsten wire (wire diameter, 25 μm; purity, 99.99%; The Nilaco Corporation, Tokyo, Japan) was used as the primary material for the tungsten oxide nanorods, and the heater, for the wire-heated environmental TEM sample holder, which enabled the introduction of gas into the environmental TEM. The holder was equipped with electrodes and the gas-introducing nozzle; the tungsten wire was connected between the electrodes and heated by current being applied to the wire. The measurement of the temperature of the heated wire was attempted using both a thermocouple and radiation thermometer. However, due to the small size of the wire, the thermocouple could not touch the wire. Furthermore, the measurement area of a radiation thermometer is larger than the wire diameter; therefore, space and material other than the wire was included in the measurement area. As a result, the wire temperature could not be measured by either the thermocouple or the radiation thermometer. Consequently, the wire temperature was measured using the following method. First, a pure metal powder with a known melting point was set on the connected wire. Secondly, the holder was introduced into the environmental TEM and the wire was heated. Then, the current was recorded when the metal powder melted; the same procedure was repeated with other metal powders. Finally, the temperature at which each metal melted was plotted on a current-temperature graph. This graph allowed us to determine the wire temperature without a thermocouple or radiation thermometer. The sample-heating holder was inserted in the environmental TEM, and the pressure in the environmental TEM was regulated by flow-rate control of the injected oxygen gas through the nozzle. The tungsten oxide nanorods grew after the current flowed through the tungsten wire. The environmental TEM used in the present study was made by HITACHI (H-9000NAR, Tokyo, Japan) and was equipped with a Gatan imaging filter [GIF] (Tokyo, Japan), a CCD, and a camera. This machine was operated at an accelerating voltage of 300 kV. The GIF was used to determine the elemental maps and electron energy loss spectra of the samples, and the dynamic growth behavior of the samples was recorded by the camera. The growth conditions used were as follows: the wire temperature was 800°C, and the oxygen pressure in the environmental TEM was 1.0 × 10^-4 ^Pa. These growth conditions were applied for all the samples grown. Moreover, the existence and shape of the grown material on the wire were observed by scanning electron microscopy [SEM] (HITACHI, S-4300). The wire was removed from the TEM holder for the SEM observations. In thick crystalline tungsten, it is difficult to observe the natural surface oxide layer on the wire and the behavior of the interface between the nanorods and wire due to the difficulty of the transmission of electrons for TEM analyses. In this case, a part of the tungsten wire was fabricated into a thin film, in which electrons can transmit through, by a focused ion beam [FIB] (JEM-9320FIB, JEOL Ltd., Akishima, Tokyo, Japan). The FIB was operated at an accelerating voltage of 20 kV.

## Results and discussion

**Figure 1:**
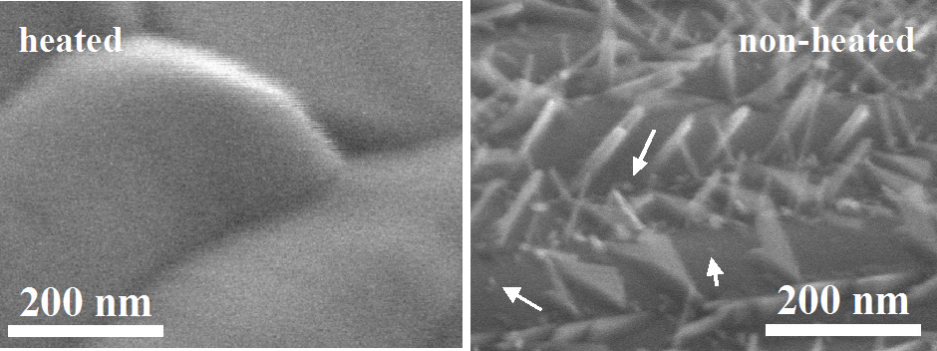
**SEM images of the heated and non-heated tungsten wire**.

SEM images of the tungsten wire that was heated in an oxygen atmosphere and the non-heated wire are shown in Figure 1; the heating time was 10 min. Both the heated and non-heated wires have asperity, which originated in the wiredrawing die when the tungsten bulk was processed from ingot to wire. In comparing the differences between the heated and the non-heated wire, it was recognized that two types of growth structures exist on the heated wire: one is nanorods with an average length and diameter of about 100 and 15 nm, respectively, while the other is a blunt angle, isosceles triangle-like plate. However, there were few of the latter structures on the wire, so the nanorods were the primary focus. The growth materials, which were located on the same asperity surface, were mutually parallel. It is proposed that the reason for this is that the direction of material growth was dependent on the bottom crystal face, which was the same as the asperity surface on the wire. In addition, nanometer-sized bright contrasts were confirmed in Figure 1, and they were showed by white arrows. It was inferred that these contrasts were nanorod and triangle-like structures during growth.

**Figure 2:**
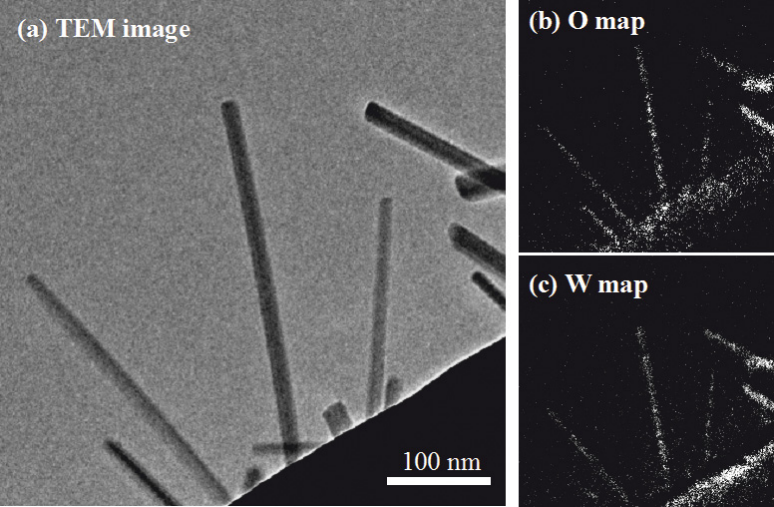
**TEM image and elemental mapping of the nanorods**. (**a**) TEM image. (**b**) O map. (**c**) W map.

Elemental mapping was carried out to examine the material of the nanorod using the energy filter of the TEM. The TEM images and oxygen and tungsten mappings are shown in Figure 2. Nanorods with diameters of about 10 to 20 nm and various lengths were confirmed in the TEM image in Figure 2a. Oxygen and tungsten mappings of Figure 2a area are shown in Figure 2b, c. The existence of tungsten and oxygen was detected in the nanorod area in Figure 2a, which confirmed that the nanorods were made from tungsten oxide. The tungsten wire was located at the base of the tungsten oxide nanorod shown in the lower right of Figure 2a.

**Figure 3:**
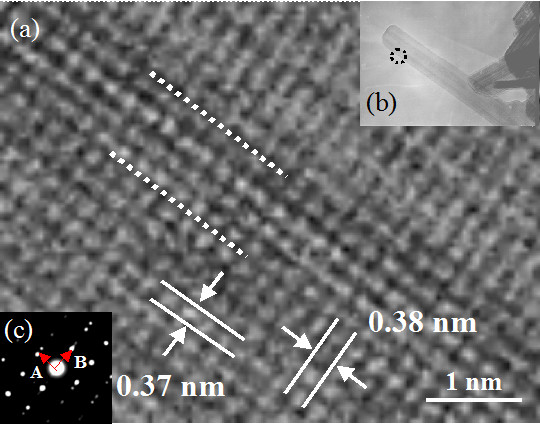
**HRTEM image (a), TEM image (b), and SAED pattern (c) of the tungsten oxide nanorod**.

High-resolution TEM [HRTEM] images and selected area electron diffraction [SAED] of the nanorods were obtained to reveal the crystal structure and orientation of the nanorod (Figure 3). The HRTEM image of the tungsten oxide nanorod (marked by a broken-line circle) in Figure 3b correlates with that in the TEM image shown in Figure 3a. Regularly aligned lattice dots, which exist along the longer directional axis and perpendicular axis against longer direction of the nanorod in Figure 3b, were confirmed in Figure 3a. Lattice dots were aligned in intervals of 0.38 nm in the longer directional axis of the nanorods and in intervals of 0.37 nm in the perpendicular direction to the longer directional axis. These interval distances of 0.38 and 0.37 nm correlate to the distances of (002) and (020) of WO_3_, which has a monoclinic crystal structure (Joint Committee on Powder Diffraction Standards [JCPDS] card no. 83-0951). These results and elemental mapping revealed that the nanorods grown from tungsten wire comprise WO_3 _with a monoclinic system. As shown in Figure 3c, two different cyclic spots existed in the SAED pattern. One cyclic spot aligned with the A vector, which correlates with the longer direction of the nanorod, and the other cyclic spot aligned with the B vector, which correlates with the direction perpendicular to the longer direction of the nanorod. The latter cyclic spot has two different brightness intensities with a weaker cyclic spot shift of the B vector from the strong cyclic spot. However, spot shift was not observed in the cyclic spot aligned with the A vector. This phenomenon indicates that dislocation exists only in the parallel plane in the longer direction of the nanorod, but not in the perpendicular plane against the longer direction of the nanorod. Dislocations were apparent in Figure 3a, as indicated by the broken white lines along the longer direction of the nanorods. It is proposed that the moderate shift of the lattice dots at the broken white line is due to dislocation.

**Figure 4:**
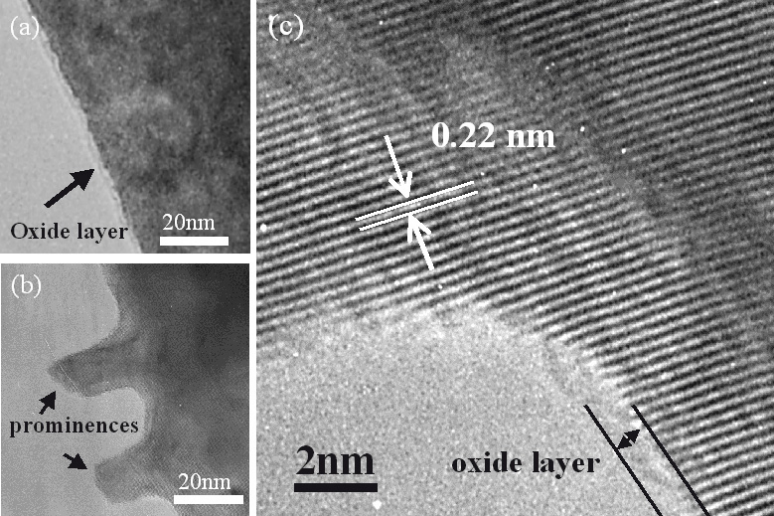
**TEM of tungsten nanowire and HRTEM and TEM images of the nanorod**. (**a**) TEM image around the surface of non-heated tungsten wire. (**b**, **c**) TEM and HRTEM images of the initial growth of the nanorod, respectively.

The above results revealed the material and structure of the nanorod. Additionally, TEM observation of the initial WO_3 _nanorod growth was conducted to investigate the growth mechanism. TEM and HRTEM images of the surface of the non-heated tungsten wire and a nanorod that was halted in the initial growth stage are shown in Figure 4. In Figure 4a, which presents the appearance of the non-heated tungsten wire surface, it is shown that a 2-nm-thick amorphous layer was coated perfectly on the surface of the tungsten wire. This thin layer was speculated to be natural tungsten oxide. In Figure 4b, which shows the surface of tungsten wire in the initial growth stage, prominences with similar widths appeared on the surface of the tungsten wire. Moreover, the result of the observed prominence root area under high magnification is shown in Figure 4c, which shows that lattice fringes with distances of 0.22 nm exist continuously in the prominences and tungsten wire. This fringe distance conforms to (110) of tungsten, as indicated in JCPDS card no. 04-0806, so the prominence is thought to be constructed of crystal tungsten. An amorphous oxide layer was observed on the surface of the tungsten wire where the nanorod did not grow, but that amorphous layer was not observed on the top of the prominence (Figure 4b). These results indicate that a heaving bottom wire was not the origin of this prominence. Furthermore, tungsten does not exist in vapor form, so the prominence could not have been formed from accumulating tungsten from vapor. Here, the process of the prominence appearing is assessed. When the tungsten wire was heated at 800°C, the wire expanded. However, the wire had an amorphous oxide layer on the surface. The thermal expansion coefficients of tungsten and amorphous tungsten oxide are about 4.5 × 10^-6 ^and 12 × 10^-6 ^[9], respectively; therefore, amorphous tungsten oxide is more likely to expand than tungsten. However, the volume of the surface amorphous oxide layer is much smaller than that of the tungsten under the surface oxide layer, so the volume expansion of tungsten is much larger than that of the amorphous oxide layer when the tungsten wire is heated. Tungsten oxide has a hardness of between 5 and 7 GPa at around 800°C [10], but the layer is fractured and thin and it is expected that cracks formed in the oxide layer. As a result, the tungsten under the oxide layer was exposed to a decompression atmosphere, forcing tungsten to diffuse through the cracks in the oxide layer by thermal expansion stress-induced diffusion and form the tungsten prominence. The reason that prominences were not formed at the area covered by the amorphous oxide layer when the wire was heated is thought to be that it is more difficult for tungsten to diffuse onto the surface when it is covered by the oxide layer. Lee et al. heated a tungsten film to 850°C to obtain a crystal tungsten nanowire with a length of over 1 μm [11]. Their growth conditions are similar to ours with the exception of the atmospheric gas. Therefore, the reason that our nanorods comprised tungsten oxide is oxidation by oxygen as the atmospheric gas. Since the prominence comprises tungsten during the initial growth stage and the atmospheric gas is oxygen, it is suggested that the tungsten prominence initially grew followed by oxidization. After that, WO_3 _nanorods were grown. If cracks occurred in the surface oxide layer when the wire was heated, the formation of the prominence by tungsten diffusing through the cracks is expected. However, tungsten, with a hardness of about 2 GPa, is very hard [10], so the possibility of prominence growth occurring via tungsten deformation only at the crack area is low. Additionally, the melting point of tungsten is 3,422°C, which is much higher than the growth process temperature of nanorods [12]. Hence, it is unlikely that tungsten evaporated through the cracks. From the TEM and HRTEM observations, it is most likely that cracks occurred in the oxide layer, and then the tungsten prominence grew through the cracks, as presented above.

**Figure 5:**
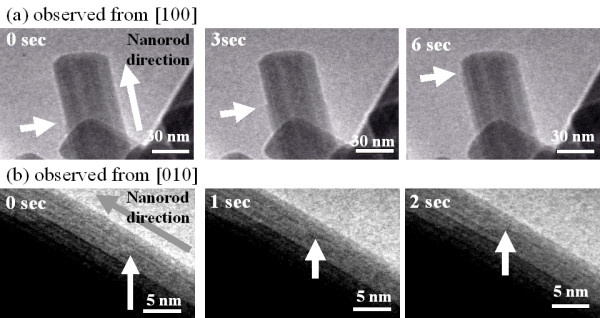
**Environmental TEM images of the growing WO_3 _nanorod observed from [100] (a) and [010] (b)**.

Environmental TEM images of the growing WO_3 _nanorod observed from [100] and [010] to reveal the WO_3 _nanorod middle growth mechanism are shown in Figure 5. Steps pointed by white arrows in Figure 5a were confirmed on the edge of the nanorods; the steps grew and moved to the top of the nanorods, as observed from the [100] direction in Figure 5a. The steps were not confirmed on the edge of the nanorod observed from the [010] direction. Instead, a changing contrast line marked by white arrows that gradually moved to the top of the nanorods was present, as shown in Figure 5b. This line was proposed to be the edge step of the nanorod observed from the [100] direction. These results indicate that the plane on (010) grows preferentially during WO_3 _nanorod growth.

The growth mechanism has often been discussed in other papers written about the growth of nanorod structures; the vapor-liquid-solid [VLS] and vapor-solid [VS] growth mechanisms are well known [13]. The VLS growth mechanism is the method in which the vapor is melted in a catalyst and then segregated. The VS mechanism is the method in which the original sources are dissolved in vapor and then crystals formed on the substrate. Catalysts are needed for VLS growth, and there were no catalysts on the top of the nanorod in Figure 5a. Therefore, the growth mechanism of the WO_3 _nanorod was not VLS. Moreover, origin gases are needed in the case of the VS mechanism. In this study, the only origin gas was oxygen, and tungsten gas was not introduced in the environmental TEM. The possibility of the evaporation of tungsten oxide, which existed originally or formed by heating in oxygen on the wire, was imagined. However, the heating temperature was 800°C, which is lower than the required 1,400°C for the evaporation of tungsten oxide [14]. As a result, the VS growth mechanism was not reasonable for the nanorod growth mechanism. In this study, the oxygen and tungsten originated from vapor and tungsten wire, respectively, so it is presumed that the tungsten, which was supplied from the tungsten wire, was oxidized by oxygen in vapor, and then WO_3 _nanorods were grown on the wire. These deliberations and results show that WO_3 _nanorods are grown from the tungsten prominence seen in Figure 4b by lateral growth.

Next, the growth of WO_3 _nanorods from the tungsten prominence is discussed. Engel et al. investigated the tungsten face that most easily absorbed oxygen and determined that the (110) face of tungsten absorbed the most oxygen [15]. Figure 4 suggests that the side edge of the prominence was (110) of tungsten and oxygen absorbed preferentially on (110). It was also inferred that WO_3 _formed preferentially on (110) of the edge of the tungsten prominence, and then oxygen absorbed on the (010) face of the WO_3 _formed on the tungsten prominence. After that, the (010) and (001) faces of WO_3_, which absorbed oxygen easily and are the closest and close-packed planes [16], grew. The origin of the tungsten is the bottom of the tungsten wire, so this acts as the tungsten supply for the nanorods. Therefore, the growth of WO_3 _on the edge of the nanorod starts from the bottom to the top of the nanorod. The reason that WO_3 _nanorod growth disappears at the area covered by the natural oxide layer when the tungsten wire was heated is that the tungsten prominence, which has the planes that easily absorb oxygen, do not grow.

In summary, the mechanism of the WO_3 _nanorod growth was determined to be as follows: cracks occurred in the surface of the natural tungsten oxide layer when the tungsten was heated, after which tungsten diffused through the cracks of natural tungsten oxide layer from the tungsten wire to form a highly crystalline prominence. The (110) plane of the tungsten prominence was preferentially oxidized to form WO_3_. Tungsten and oxygen are supplied to the WO_3 _surface from the bottom tungsten wire and atmosphere, respectively, resulting in continual growth of the WO_3 _nanorods. To obtain further evidence for the proposed growth mechanism, a part of the oxide layer on the tungsten substrate needs to be fine-fabricated by FIB, electron beam lithography, etc., and then heated in an oxygen atmosphere, and the appearance of WO_3 _nanorod growth will have to be confirmed.

## Conclusions

WO_3 _nanorods were grown by heating a tungsten wire in an oxygen atmosphere, and the growth of WO_3 _was observed by environmental TEM and HRTEM. In particular, the initial and the middle growth were observed. The growth mechanism involving the initial formation of cracks in the surface natural oxide layer on the tungsten wire followed by the formation of a tungsten prominence that was subsequently oxidized to form the WO_3 _nanorods was proposed. The tungsten and oxygen were supplied from the tungsten wire and the oxygen atmosphere, respectively. WO_3 _nanorod growth was suggested by TEM observation.

## Competing interests

This work was supported by a Grant-in-Aid for Young Scientists (B program, no. 22760537) and a research grant from the Murata Science Foundation.

## Authors' contributions

TT carried out the TEM observation with TK and KT, and drafted the manuscript. TK and KT controlled the environmental condition in environmental TEM when the sample was observed. NN carried out the sample preparation by FIB. YH participated in the design of the sample preparation. KS performed the heater calibration and maintained the environmental TEM. KK participated in the study design. TY coordinated this work. All authors read and approved final manuscript.
